# Interleukin‐3 Modulates Macrophage Phagocytic Activity and Promotes Spinal Cord Injury Repair

**DOI:** 10.1111/cns.70181

**Published:** 2024-12-19

**Authors:** Jianjian Li, Meige Zheng, Fangru Ouyang, Jianan Ye, Jinxin Huang, Yuanzhe Zhao, Jingwen Wang, Fangli Shan, Ziyu Li, Shuishen Yu, Fei Yao, Dasheng Tian, Li Cheng, Juehua Jing

**Affiliations:** ^1^ Department of Orthopaedics The Second Affiliated Hospital of Anhui Medical University Hefei China; ^2^ Institute of Orthopaedics, Research Center for Translational Medicine The Second Affiliated Hospital of Anhui Medical University Hefei China

**Keywords:** astrocyte, IL‐3, IL‐3Rα, lipid droplets, macrophage, SCI

## Abstract

**Background:**

Effective clearance of lipid‐rich debris by macrophages is critical for neural repair and regeneration after spinal cord injury (SCI). Interleukin‐3 (IL‐3) has been implicated in programming microglia to cluster and clear pathological aggregates in neurodegenerative disease. Yet, the influence of IL‐3 on lipid debris clearance post‐SCI is not well characterized.

**Methods:**

We established a mouse model of spinal cord compression injury to investigate the role of IL‐3. Blockage of IL‐3 was achieved through intrathecal delivery of an IL‐3‐neutralizing antibody, while IL‐3 activation was augmented via in situ injection of recombinant IL‐3 into the lesion site immediately post‐SCI. Immunofluorescence staining was performed to determine IL‐3 and IL‐3Rα sources and distribution, lipid droplet accumulation, neuron preservation, and axon regeneration after SCI. The Basso Mouse Scale (BMS) and footprint analysis were employed to evaluate locomotor function recovery.

**Results:**

We found that IL‐3 expression was significantly upregulated post‐SCI, peaking at 14 days post‐injury (dpi) and persisting until 28 dpi. Notably, IL‐3 was primarily secreted by astrocytes surrounding the lesion epicenter. Correspondingly, IL‐3Rα was predominantly observed in macrophages within the injury core, also elevating at 14 dpi. Neutralization of IL‐3 led to increased lipid droplet accumulation, along with markedly widespread of macrophages and decreased neuronal survival, resulting in severe motor deficits compared to controls. Conversely, in situ injection of IL‐3 reduced lipid droplet accumulation in macrophages, preserved neurons, promoted axon regeneration, and ultimately contributed to the recovery of motor function after SCI.

**Conclusion:**

Our findings shed light on the role of IL‐3 in modulating macrophage phagocytic activity and suggest that the IL‐3/IL‐3Rα pathway may be a potential therapeutic target for enhancing neural repair and functional recovery after SCI.

## Graphical abstract

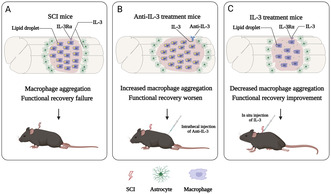

## 1 Introduction

The aftermath of spinal cord injury (SCI) is characterized by extensive cell death, giving rise to a lipid‐rich environment due to the accumulation of cellular and myelin debris [1, 2]. This milieu is associated with the formation of pro‐inflammatory foamy macrophages [1, 3], which contribute to chronic inflammation and neurological deterioration [4, 5]. Moreover, myelin‐activated macrophages are implicated in axonal dieback and retraction [6], a phenomenon that underscores the therapeutic potential of modulating macrophage lipid metabolism after SCI [2, 7, 8]. In response to SCI, astrocytes undergo rapid proliferation, migration, and form astrocytic scars, which serve to compact the inflammatory macrophages and contract the lesion area [9, 10, 11, 12]. However, the intricate interaction between astrocytes and macrophages in the SCI context is not fully understood.

Interleukin‐3 (IL‐3) is a hematopoietic growth factor with multifaceted roles in inflammation [13]. In the central nervous system (CNS), IL‐3 levels correlate with the pathology of neurodegenerative diseases [14, 15, 16]. Although IL‐3 has been extensively studied for its cytokine functions [17, 18, 19, 20], recent investigations have unveiled the IL‐3/IL‐3Rα signaling pathway as a mediator of astrocyte–macrophage communication, exacerbating conditions such as multiple sclerosis [21]. Additionally, in Alzheimer's disease, astrocytic IL‐3 activates microglia through the IL‐3Rα receptor, initiating the clearance of β‐amyloid and tau aggregates [22]. These findings highlight the critical role of IL‐3/IL‐3Rα pathway in neuroinflammatory diseases. However, the specific actions of IL‐3 in SCI remain undefined.

Here, we observed a significant upregulation of IL‐3 distributing around the injury core after SCI, with astrocytes identified as the primary source. Concurrently, IL‐3Rα expression in macrophages within the injury core was enhanced. Neutralization of IL‐3 impeded lipid droplet clearance by macrophages, while in situ IL‐3 administration facilitated this process, supporting neuronal preservation, axon regeneration, and leading to motor function recovery after SCI. These results highlight the essential role of the IL‐3/IL‐3Rα pathway in lipid clearance and suggest a potential crosstalk pattern between astrocytes and macrophages after SCI.

## 2 Methods and Materials

### 2.1 Animals

C57BL/6J mice were obtained from the Experimental Animal Center of Anhui Medical University and were housed under standardized conditions, including controlled temperature and humidity, as well as a 12:12 h light–dark cycle. All mice included in this study were adult female individuals aged between 8 and 12 weeks old at the time of SCI induction. Ethical approval for all experimental procedures involving the mice was granted by the Institutional Animal Ethical Committee of Anhui Medical University (Approval No. LLSC20231692). The mice were randomly assigned to different experimental groups and were provided with ad libitum access to both food and water in their individual cages.

### 2.2 SCI Model

All surgical procedures were performed under anesthesia using ketamine (100 mg/kg) and xylazine (15 mg/kg). In brief, upon a single laminectomy, the T10 spinal cord was cautiously exposed and compressed entirely for a duration of 5 s using Dumont #5 forceps (11252‐20, Fine Science Tools, Germany). Subsequently, the mice were allowed to fully regain consciousness and were randomly assigned numerical identifiers. Throughout the study, the mice received twice‐daily assistance with urination.

### 2.3 Intrathecal Injection of IL‐3‐Neutralizing Antibody

The needle insertion site for intrathecal injection was localized at the dorsal center of the lumbar 5–6 intervertebral space, as previously described [23]. A total volume of 2 μL, containing IL‐3‐neutralizing antibody (MAB403, R&D Systems) at a concentration of 100 ng/μL, dissolved in sterile phosphate‐buffered saline (PBS, Servicebio, China), was administered at a rate of 1 μL/30 s using a microinjection needle (1701, Hamilton, Switzerland). Confirmation of successful needle penetration into the intradural space was achieved by the observation of a sudden tail flick response. This intrathecal injection was performed under anesthesia once daily, starting from 4 h post‐injury until 14 days post‐injury (dpi). In the control group, the mice were subjected to an equal volume of rat IgG (16‐4301‐81, Invitrogen).

### 2.4 In Situ Injection of IL‐3

The T10 spinal cord was exposed following the established protocol for inducing the SCI model as described above. Subsequently, the mouse was immobilized using a stereotaxic apparatus. The precise insertion site was determined to be 0.3 mm lateral to the midline and 0.8 mm deep from the dorsal surface of the spinal cord in accordance with previous study [24]. A total of 200 ng of recombinant mouse IL‐3 (HY‐P73207, MCE), dissolved in 2 μL of sterile PBS was injected into the injured spinal cord immediately after the SCI procedure, at a controlled rate of 0.4 μL/min using a stereotaxic injector (KDS LEGATO 130, RWD, China). The control group received an equal volume of sterile PBS. At the end of the experiment, all mice were sacrificed at 28 dpi for subsequent analysis.

### 2.5 Histology and Immunofluorescence Staining

After transcardiac perfusion with PBS followed by 4% paraformaldehyde (Servicebio, China), spinal cord tissues were dissected with the injury site as the central point, spanning a total length of 8 mm. The tissues were post‐fixed overnight in 4% paraformaldehyde, dehydrated in a 30% sucrose solution for 48 h and then cut into 16 μm‐thick serial sagittal sections using a cryostat (NX50, Thermo Fisher Scientific, United States). Frozen sections were prepared for immunofluorescence as described [23]. The primary antibodies used in this study were as follows: rat anti‐IL‐3 (1:10, 503902, Biolegend), rabbit anti‐IL‐3Rα (1:100, 141039, US Biological), goat anti‐CD31 (1:200, AF3625, R&D Systems), rat anti‐F4/80 (1:100, 14‐4801‐82, Invitrogen), goat anti‐PDGFRβ (1:100, AF1042‐SP, R&D Systems), rat anti‐GFAP (1:200, 13‐0300, Invitrogen), rabbit anti‐GFAP (1:100, 16825‐1‐AP, Proteintech), goat‐Iba1 (1:200, NB100‐1028, Novus), rat anti‐CD68 (1:300, MCA1957, AbD Serotec), goat anti‐5‐HT (1:5000, #20080, Immunostar), and rabbit anti‐NeuN (1:500, ab177487, Abcam). The corresponding secondary antibodies used were as follows: Alexa Fluor 488 (1:500, A‐21208, A‐21206, A‐11055, Invitrogen) and Alexa Fluor 555 (1:500, A‐31572, A‐48270, A‐21432, Invitrogen). BODIPY 493/503 (1:1000, P0051, DuoFlour, China) was used for lipid droplet staining. 4′,6‐Diamidino‐2‐phenylindole, dilactate (DAPI, P0126, Beyotime Biotechnology, China) was used for nuclear staining. Immunofluorescent images were examined and photographed using a fluorescence microscope (Axio Scope A1, Zeiss, Germany). Images were processed by Zen 3.1 software (Blue edition).

### 2.6 Quantitative Analysis

To assess the areas of GFAP^+^ and IL‐3^+^ immunoreactivity, the immunostaining signals were normalized to the area of the spinal cord segment encompassing the injured core in a 4× magnification image. The relationship between GFAP and IL‐3 positive areas was analyzed by using GraphPad Prism 7.0 software (GraphPad, United States) for correlation assessment. Three animals per group with 3–5 sections per animal were analyzed for this measurement.

To evaluate the ratio of IL‐3^+^ astrocytes relative to the total number of astrocytes, IL‐3^+^GFAP^+^ astrocytes and GFAP^+^ astrocytes were quantified in three randomly captured images at 40× magnification. Similarly, the ratio of IL‐3^+^ cells relative to different cell types including CD31^+^ endothelial cells, PDGFRβ^+^ fibroblasts, CD68^+^ macrophages/microglia, and NG2^+^ cells, were counted in three randomly selected 40× magnification images of the injured core. To evaluate the ratio of IL‐3Rα^+^ macrophages relative to the total number of macrophages, IL‐3Rα^+^IBA1^+^, IL‐3Rα^+^MAC2^+^, IL‐3Rα^+^F4/80^+^, IBA1^+^, MAC2^+^, and F4/80^+^ cells were counted in three randomly captured images at 40× magnification. Three samples per group with 3–5 sections per animal were examined for this quantification. Only DAPI^+^ cells were included in the counting.

To assess BODIPY^+^, MAC2^+^, and CD68^+^ areas, ImageJ/Fiji software (version 2.3.011/1.53f51) was used, as previously described [23], in 4× magnification images. To evaluate the areas of injury, the immunoreactivity of GFAP^−^ area was normalized to the area of the spinal cord segment spanning the injured core in a 4× magnification image. Four to five samples per group with 3–5 sections per animal were analyzed for this measurement.

To quantify the regenerated 5‐HT axons growing at the injured site, the distance from the tip of the 5‐HT axon to the edge of the astrocytic scars was measured using ImageJ/Fiji software. Four to five samples per group with 3–5 sections per animal were included in this analysis. To quantify the number of NeuN^+^ cells, the sagittal sections were divided into three zones based on their proximity to the core of the lesion, as previously described: Z1 (0–250 μm), Z2 (250–500 μm), and Z3 (1000–1250 μm) [25]. The number of NeuN^+^ cells was counted using ImageJ/Fiji software.

### 2.7 Motor Behavioral Analysis

All mice underwent a standardized test adaptation protocol 1 h prior to the behavioral analysis [26]. The assessment was conducted in a double‐blind fashion by two highly experienced and independent researchers. Basso Mouse Scale (BMS) scores were evaluated before SCI and at 7, 14, and 28 dpi utilizing a well‐established open field rating scale. The scoring system ranged from 0 to 9, encompassing a comprehensive spectrum for motor function assessment. To ensure robustness and objectivity, the final score for each mouse was determined as the mean of the scores assigned by the two investigators. To further elucidate hind limb locomotion, footprint analysis was performed at 28 dpi [27]. Following the application of contrasting paint to the fore and hind limbs, the mouse traversed a precisely constructed channel, measuring 70 cm in length and 5 cm in width. Subsequently, gait analysis was conducted to evaluate various parameters, including stride length, stride width, and paw rotation.

### 2.8 Statistical Analysis

Data were presented as mean ± standard error of the mean (SEM). Individual data points were shown in the figures. The statistical methods used were described in the figure legends. The Shapiro–Wilk test was utilized to assess data distribution for normality. Multiple comparisons were performed using one‐way or two‐way analysis of variance (ANOVA) with Tukey's or Bonferroni's post hoc test. Comparisons between two groups were performed using Student's *t*‐test. Data analysis and graph generation were performed using GraphPad Prism 7.0 (GraphPad, United States), and a value of *p* < 0.05 was regarded as statistically significant.

## 3 Results

### 3.1 IL‐3 Exhibits a Gradual Upregulation Following SCI and Is Predominantly Expressed by Astrocytes Surrounding the Lesion Epicenter

**FIGURE 1:**
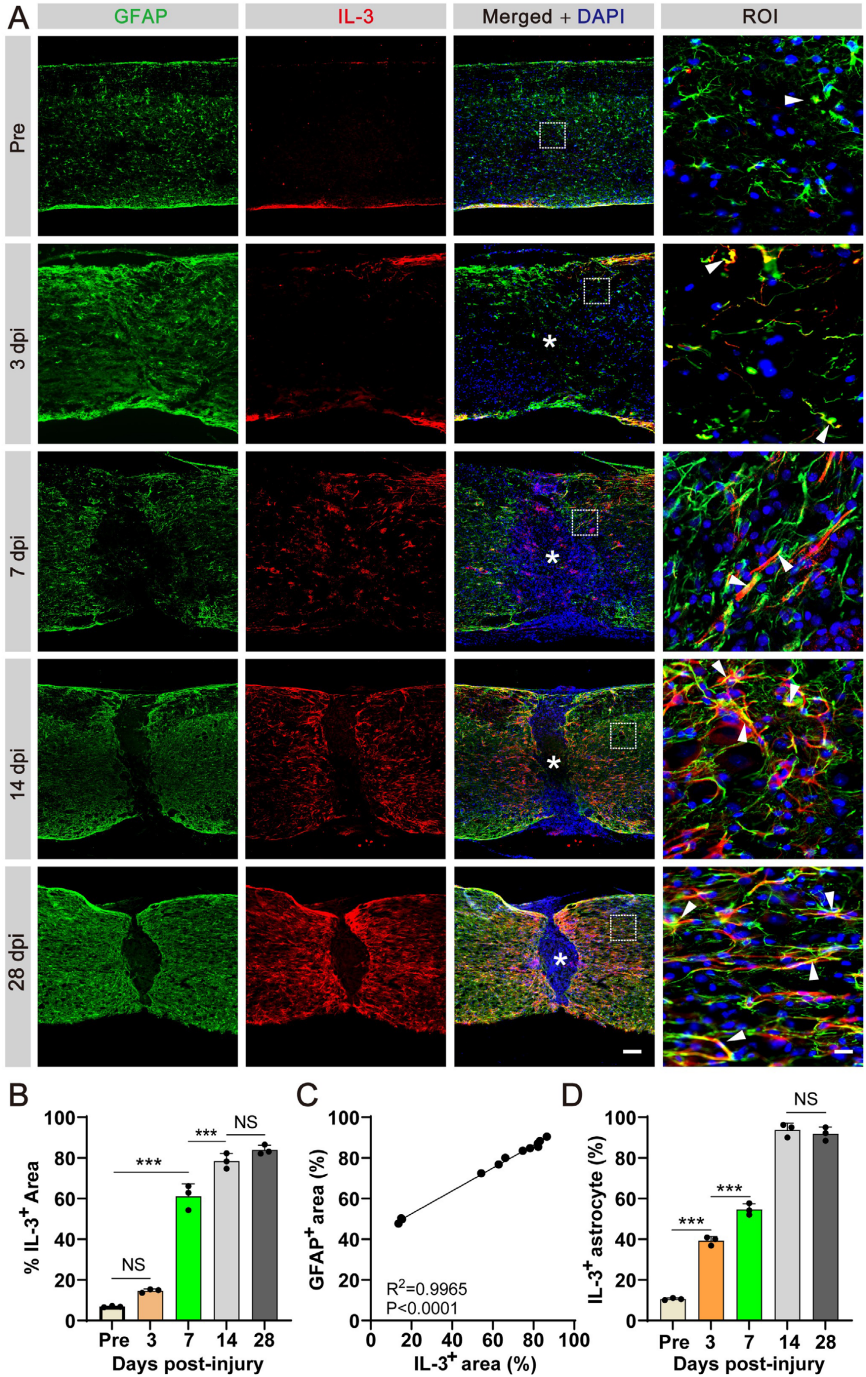
IL‐3 is gradually upregulated after SCI. (A) Spatiotemporal distribution of IL‐3 after SCI. Immunofluorescent staining of GFAP (green), IL‐3 (red) and nuclei (blue) in sagittal sections before SCI and at 3, 7, 14, and 28 dpi. The region of interest (ROI) represents the boxed region on the left. Arrowheads indicate IL‐3^+^ cells. Asterisks indicate the injured core. Scale bars: 200 μm (left panel) and 20 μm (right panel). Pre, before SCI. (B) Quantification of the percentage of IL‐3^+^ area. (C) Correlations between the percentage of GFAP^−^ area and IL‐3^+^ area scores were analyzed by Pearson correlation coefficients. (D) Quantification of the proportion of IL‐3^+^ astrocytes. *n* = 3 per time point. NS, no significance; ***p < 0.001 by one‐way ANOVA followed by Tukey's post hoc test.

Utilizing immunofluorescent staining of IL‐3 and GFAP, we traced IL‐3 expression and distribution from pre‐injury through 28 dpi. The findings indicated a progressive increase in IL‐3 levels from 3 dpi, peaking at 14 dpi and persisting until 28 dpi outside the lesion core, where it colocalized with GFAP^+^ astrocytes (Figure 1A,B). Meanwhile, IL‐3 exhibited a spatiotemporal distribution pattern similar to those reactive astrocytes and displayed a robust correlation with astrocytic localization after SCI (*R* ^2^ = 0.9965, *p* < 0.0001, Figure 1A,C). Quantitative analysis revealed that the percentage of IL‐3^+^GFAP^+^ cells relative to the total number of GFAP^+^ cells in the injured spinal cord rose to 39.25 ± 2.41% at 3 dpi, 54.62 ± 2.62% at 7 dpi, 93.73 ± 2.46% at 14 dpi, and 91.86 ± 3.14% at 28 dpi, respectively (Figure 1A,D). Notably, IL‐3 expression at 14 dpi was ~9‐fold higher compared to uninjured mice, suggesting its involvement in the pathophysiology of SCI.

**FIGURE 2:**
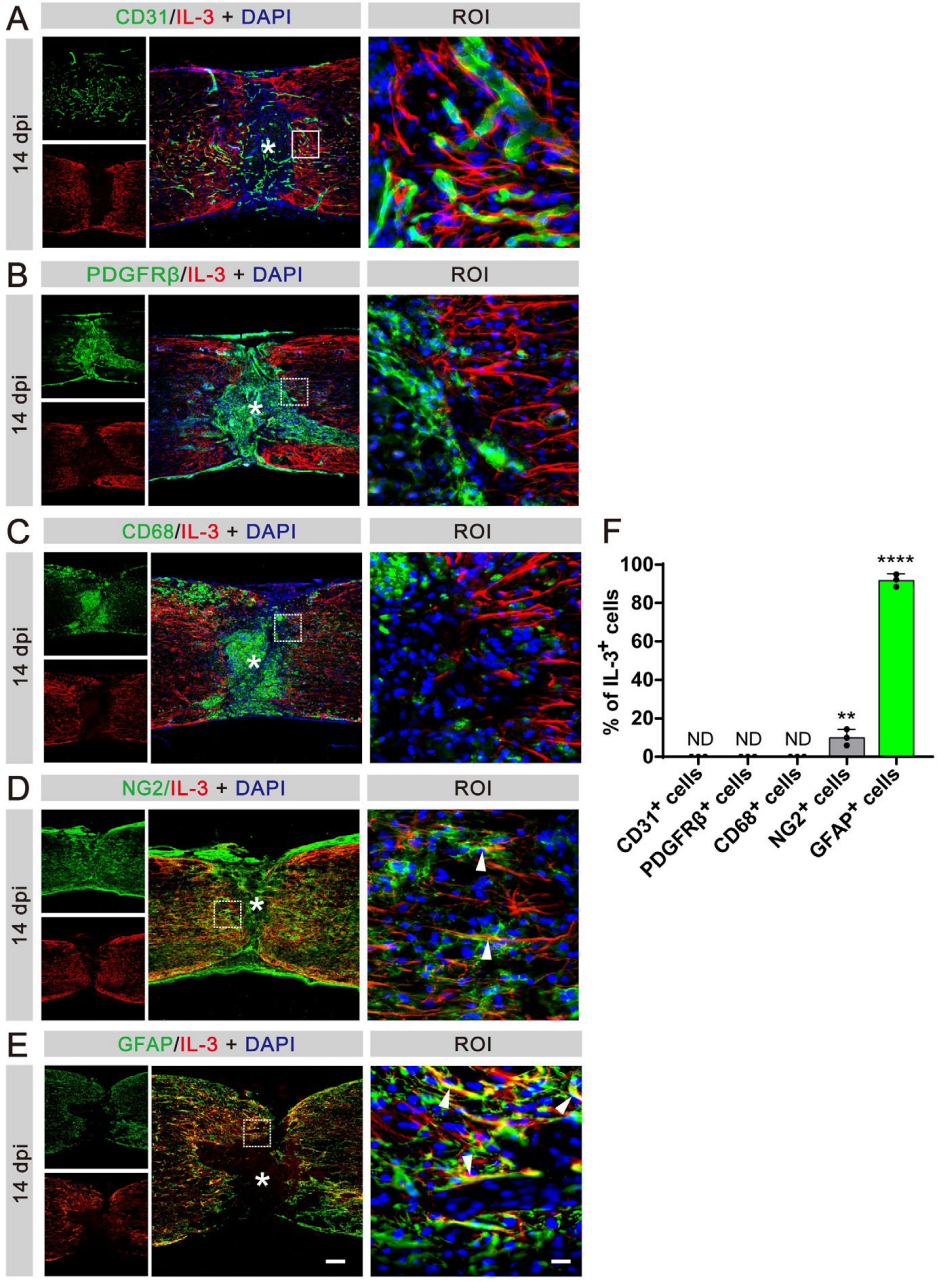
IL‐3 is specifically expressed in astrocytes at 14 dpi. (A) Immunofluorescent staining of CD31 (green), IL‐3 (red), and nuclei (blue) in sagittal sections at 14 dpi. (B) Immunofluorescent staining of PDGFRβ (green), IL‐3 (red), and nuclei (blue) in sagittal sections at 14 dpi. (C) Immunofluorescent staining of CD68 (green), IL‐3 (red), and nuclei (blue) in sagittal sections at 14 dpi. (D) Immunofluorescent staining of NG2 (green), IL‐3 (red), and nuclei (blue) in sagittal sections at 14 dpi. (E) Immunofluorescent staining of GFAP (green), IL‐3 (red), and nuclei (blue) in sagittal sections at 14 dpi. The region of interest (ROI) represents the boxed region on the left. Asterisks indicate the injured core. Scale bars: 200 μm (left panel) and 20 μm (right panel). *n* = 3 animals per group. (F) Quantification of the proportion of IL‐3^+^CD31^+^ cells in CD31^+^ cells, IL‐3^+^PDGFRβ^+^ cells in PDGFRβ^+^ cells, IL‐3^+^CD68^+^ cells in CD68^+^ cells, IL‐3^+^NG2^+^ cells in NG2^+^ cells, or IL‐3^+^GFAP^+^ cells in GFAP^+^ cells at 14 dpi. ND, no determined, **p < 0.01; ****p < 0.001 by one‐way ANOVA followed by Tukey's post hoc test.

To further validate the sources of IL‐3 post‐SCI, we examined the main cell components at the injury site. We did not observe colocalization between IL‐3 and CD31^+^ vascular endothelial cells, PDGFRβ^+^ fibroblasts, and CD68^+^ microglia/macrophages (Figure 2A–C,F). However, there was substantial co‐localization between IL‐3 and GFAP^+^ astrocytes. Additionally, a limited co‐localization was noted between IL‐3 and NG2^+^ astrocytes/oligodendrocytes, further supporting the notion that astrocytes were the primary sources of IL‐3 (Figure 2D–F). These results collectively reveal that IL‐3 expression is upregulated after SCI and peaks at 14 dpi, with astrocytes identified as the primary contributors.

### 3.2 IL‐3Rα Is Predominantly Expressed in Macrophages After SCI

**FIGURE 3:**
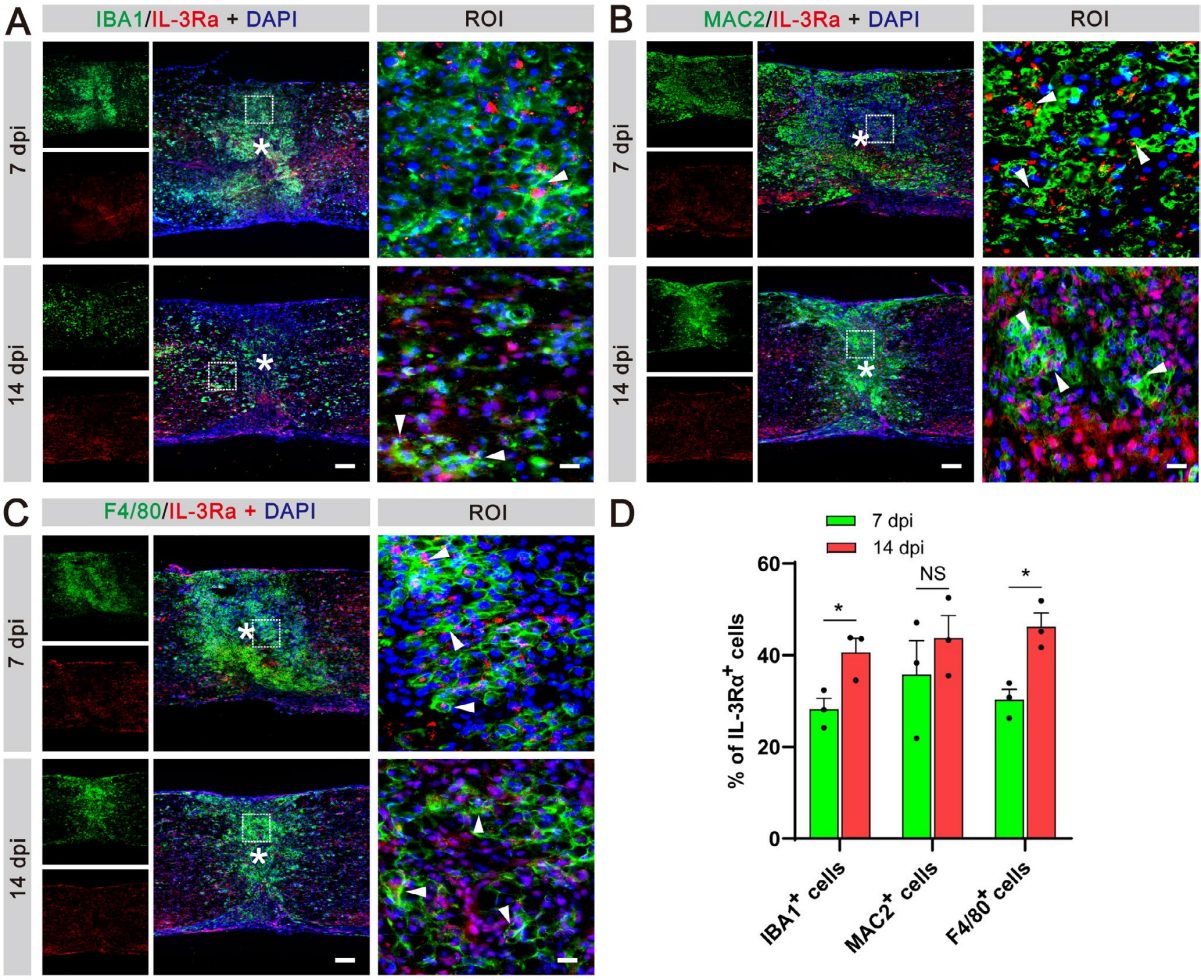
IL‐3Rα is prominently expressed in macrophages after SCI. (A) Immunofluorescent staining of IBA1 (green), IL‐3Rα (red), and nuclei (blue) in sagittal sections at 7 and 14 dpi. (B) Immunofluorescent staining of MAC2 (green), IL‐3Rα (red) and nuclei (blue) in sagittal sections at 7 and 14 dpi. (C) Immunofluorescent staining of F4/80 (green), IL‐3Rα (red), and nuclei (blue) in sagittal sections at 7 and 14 dpi. Scale bars: 200 μm (left panel in A, B, C) and 20 μm (right panel in A, B, C). *n* = 3 animals per group. (D) Quantification of the proportion of IBA1^+^IL‐3Rα^+^ cells in IBA1^+^ cells, MAC2^+^IL‐3Rα^+^ cells in MAC2^+^ cells or F4/80^+^IL‐3Rα^+^ cells in F4/80^+^ cells at 7 and 14 dpi. 14 dpi compared with 7 dpi, NS, no significance; *p < 0.05 by Student's *t* test.

After establishing astrocytes as the primary sources of IL‐3 following SCI, we aimed to identify the specific cells responsive to this cytokine. IL‐3Rα, also known as CD123, serves as the specific receptor for IL‐3. Previous studies have indicated that infiltrating myeloid cells, particularly macrophages, express IL‐3Rα in the CNS [21]. Therefore, we performed immunofluorescent staining to validate the expression of IL‐3Rα in myeloid cells following SCI, using IBA1 to label microglia/macrophages [28]. Analysis revealed that the percentage of co‐stained IL‐3Rα^+^IBA1^+^ microglia/macrophages, relative to the total number of IBA1^+^ microglia/macrophages in the injured spinal cord, was 28.23 ± 4.07% at 7 dpi and 40.64 ± 3.20% at 14 dpi (Figure 3A,D). Furthermore, we investigated the co‐expression of IL‐3Rα with MAC2 or F4/80, specific markers of macrophages [29, 30]. The percentage of co‐stained IL‐3Rα^+^MAC2^+^ macrophages relative to the total population of MAC2^+^ macrophages in the spinal cord was 35.78 ± 11.37% at 7 dpi and 43.79 ± 8.25% at 14 dpi (Figure 3B,D). Similarly, the percentage of co‐stained IL‐3Rα^+^F4/80^+^ macrophages relative to the total number of F4/80^+^ macrophages in the injured spinal cord was 30.35 ± 3.60% at 7 dpi and 46.25 ± 4.52% at 14 dpi (Figure 3C,D). Consequently, these results demonstrate that microglia/macrophages upregulate IL‐3Rα expression after SCI and may serve as responsive target cells for astrocyte‐derived IL‐3.

### 3.3 Blockage of IL‐3 Exacerbates Lipid Droplet Accumulation and Inflammation After SCI

**FIGURE 4:**
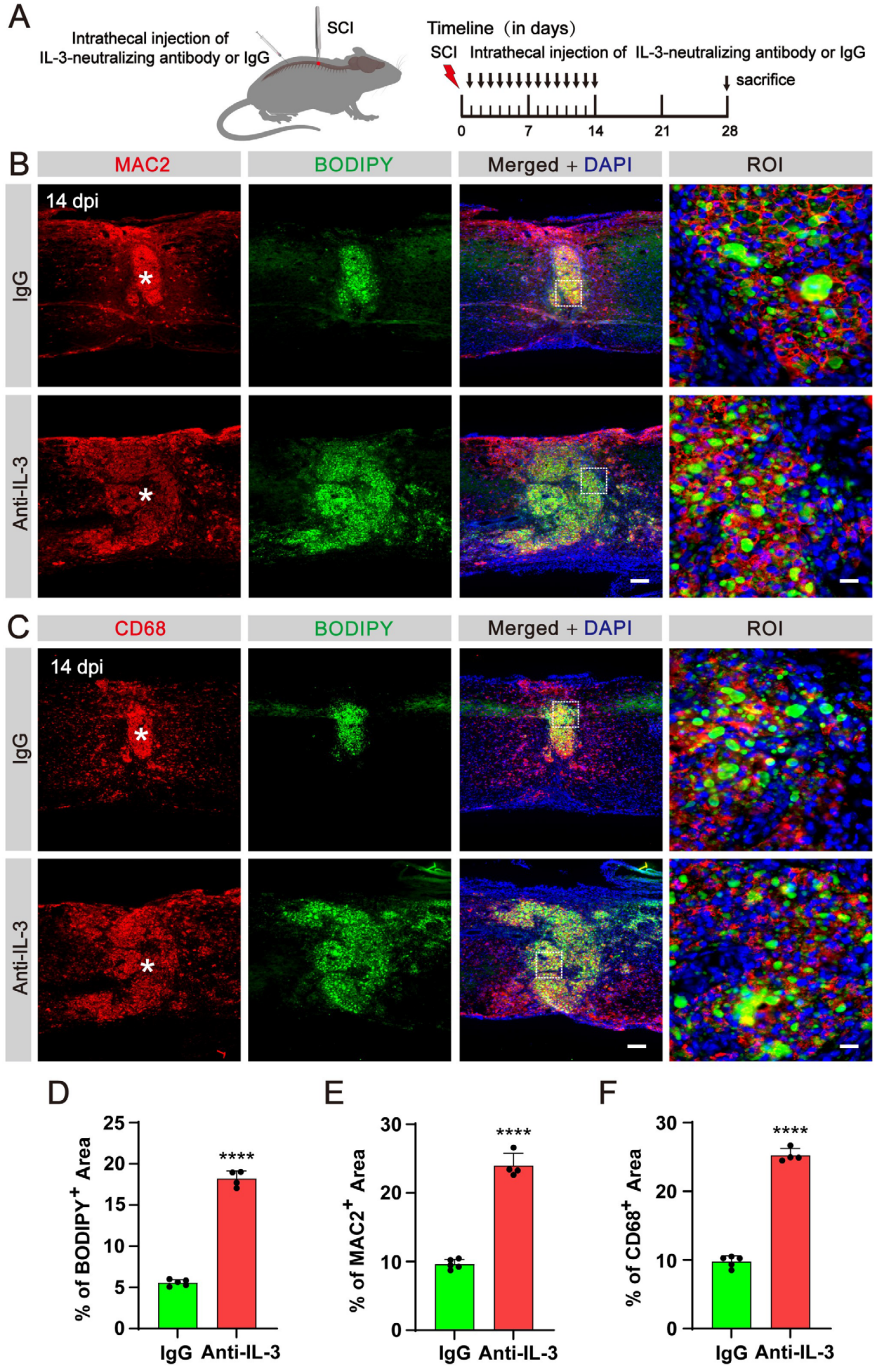
Intrathecal injection of IL‐3‐neutralizing antibody obstacles lipid droplet clearance after SCI. (A) Timeline of the intrathecal injection. (B) Immunofluorescent staining of MAC2 (red) and BODIPY (green) in sagittal sections of the IgG and Anti‐IL‐3 groups at 14 dpi. (C) Immunofluorescent staining of CD68 (red) and BODIPY (green) in sagittal sections of the IgG and Anti‐IL‐3 groups at 14 dpi. The region of interest (ROI) represents the boxed region on the left. Asterisks indicate the injured core. Scale bars: 200 μm (left panel in B, C) and 20 μm (right panel in B, C). *n* = 4–5 animals per group. (D–F) Quantification of the percentage of the BODIPY^+^ area (D), MAC2^+^ area (E) and CD68^+^ area (F) in the spinal cord segment spanning the injured core at 14 dpi. ****p < 0.0001 by Student's *t* test.

It has been previously reported that IL‐3 promotes the clustering of microglia expressing IL‐3Rα to clear pathological aggregates in neurodegenerative diseases [22]. To investigate the impact of IL‐3 on the clearance of lipid droplets and inflammation following SCI, we administered daily intrathecal injections of an IL‐3‐neutralizing antibody (anti‐IL‐3) from 1 dpi until 14 dpi to block the IL‐3/IL‐3Rα pathway (Figure 4A). We observed a decrease in IL‐3 levels in the injured spinal cord following the delivery of anti‐IL‐3, confirming the effectiveness of the neutralizing antibody (Figure S1). Immunofluorescence staining revealed a significant accumulation of BODIPY^+^ lipid droplets in macrophages at 14 and 28 dpi following intrathecal injection of anti‐IL‐3 compared to the IgG control group (Figure 4B–D, Figure S3A–C). Furthermore, blockage of IL‐3 resulted in a notable increase in the areas occupied by MAC2^+^ and CD68^+^ inflammatory cells at 14 and 28 dpi, in comparison to the IgG group (Figure 4B,C,E,F, Figure S3A,B,D,E). These findings indicate that IL‐3 blockage may impede the clearance of lipid droplets by macrophages and exacerbate inflammation in the lesion core after SCI.

### 3.4 Blockage of IL‐3 Impedes Tissue Healing, Neuronal Survival, Axon Regeneration, and Motor Function Recovery After SCI

**FIGURE 5:**
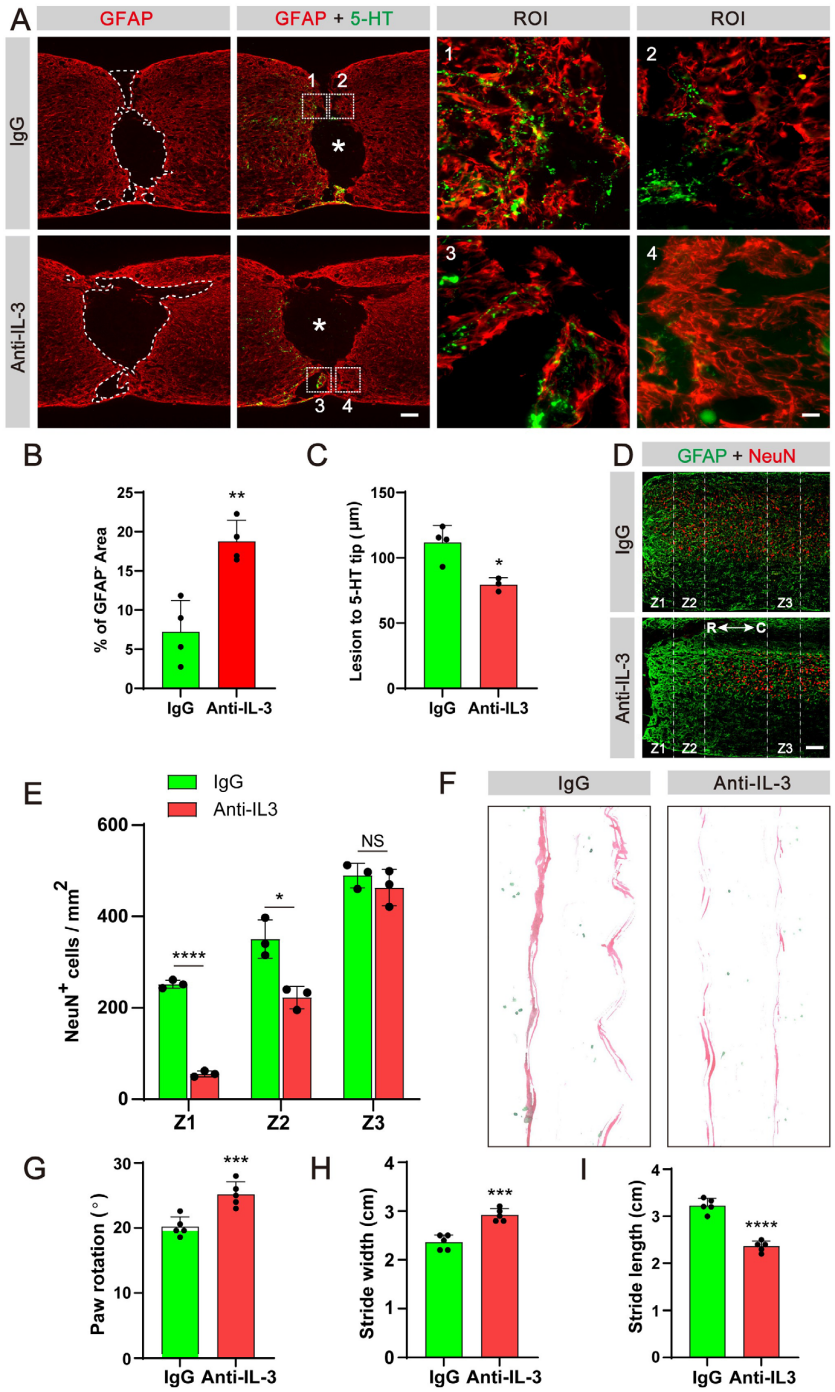
Intrathecal injection of IL‐3‐neutralizing antibody contributes to neurological decline and hinders motor function recovery. (A) Immunofluorescent staining of GFAP (green) and 5‐HT (red) in sagittal sections of the IgG and Anti‐IL‐3 groups at 28 dpi. The region of interest (ROI) represents the boxed region on the left and shows the astrocytic scar boundary. Asterisks indicate the injured core. (B) Quantification of the percentage of GFAP^−^ area in the spinal cord segment spanning the injured core at 28 dpi. (C) Quantification of the lesion distance to the 5‐HT axon tip. *p < 0.05 and **p < 0.01 by Student's *t* test, *n* = 3–4 animals per group. (D) Immunofluorescent staining of GFAP (green) and NeuN (red) in sagittal sections of the IgG and Anti‐IL‐3 groups at 28 dpi. Scale bars: 200 μm (left panel in A) and D, 20 μm (right panel in A). (E) Quantification of NeuN^+^ cells in Z1–Z3 zones adjacent to the lesion core at 28 dpi. Anti‐IL‐3 group compared with IgG group, NS, no significance, *p < 0.05 and ****p < 0.0001 by Student's *t* test, *n* = 3 animals per group. (F) Representative images of footprint analysis in the PBS and IL‐3 groups at 28 dpi. The front paws are shown in green dyes, and the hind paws are shown in red dyes. (G–I) Quantification of the paw rotation, stride width and stride length at 28 dpi. ***p < 0.001 and ****p < 0.0001 by Student's *t* test, *n* = 5 animals per group.

We further investigated the impact of IL‐3 blockage on neurological outcomes following SCI. Our findings demonstrated that mice receiving anti‐IL‐3 exhibited a larger injury area, as evidenced by the GFAP^−^ area, compared to the IgG group (Figure 5A,B). Furthermore, the anti‐IL‐3 group displayed fewer 5‐HT axonal fibers at the lesion site in comparison to the IgG group (Figure 5A,C). Additionally, the number of surviving NeuN^+^ neurons in the Z1–Z3 region of the spinal cord was significantly reduced in mice treated with anti‐IL‐3 compared to the IgG group (Figure 5D,E). This seems to be inversely correlated with the expansion of the BODIPY^+^ region, as evidenced by the decrease in NeuN^+^ signals alongside an increase in BODIPY signals (Figure S2). Moreover, analysis of footprints revealed that mice receiving anti‐IL‐3 exhibited impaired locomotor function at 28 dpi, characterized by a shorter stride length, longer stride width, and increased paw rotation compared to the mice in the IgG group (Figure 5F–I). These results suggest that pharmacological inhibition of IL‐3/IL‐3Rα signaling pathway exacerbates the expansion of trauma regions, impedes axonal regeneration, and compromises neuronal viability, culminating in pronounced motor dysfunction following SCI.

### 3.5 In Situ Injection of IL‐3 Promotes Lipid Droplets Clearance and Attenuates Inflammation After SCI

**FIGURE 6:**
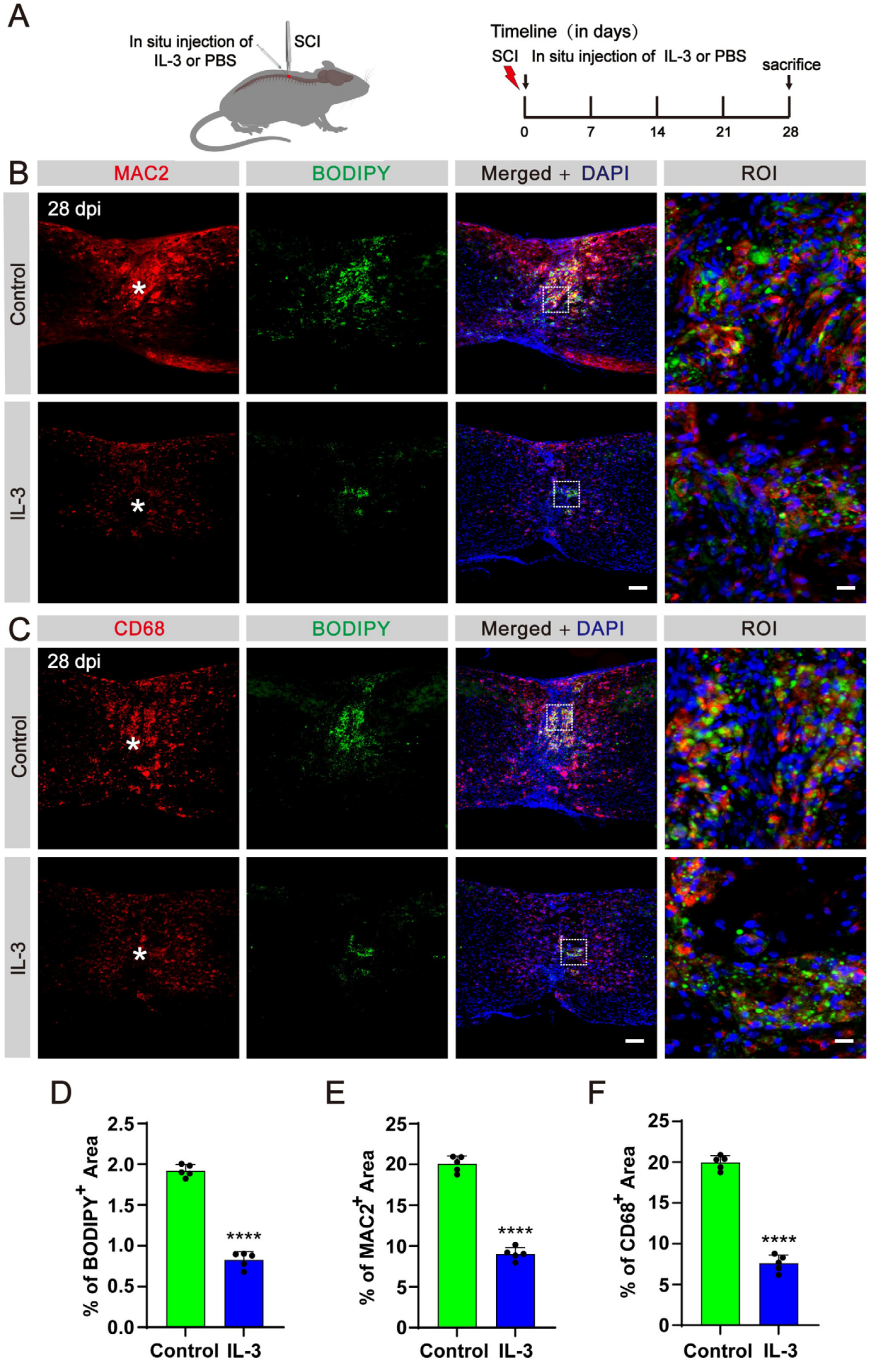
In situ injection of IL‐3 promotes the clearance of lipid droplets after SCI. (A) Timeline of the in situ injection. (B) Immunofluorescent staining of MAC2 (red) and BODIPY (green) in sagittal sections of the control and IL‐3 groups at 28 dpi. (C) Immunofluorescent staining of CD68 (red) and BODIPY (green) in sagittal sections of the control and IL‐3 groups at 28 dpi. The region of interest (ROI) represents the boxed region on the left. Asterisks indicate the injured core. Scale bars: 200 μm (left panel in B, C) and 20 μm (right panel in B, C). *n* = 5 animals per group. (D–F) Quantification of the percentage of BODIPY^+^ area (D), MAC2^+^ area (E), and CD68^+^ area (F) in the spinal cord segment spanning the injured core at 28 dpi. ****p < 0.0001 by Student's *t* test.

To validate the potential therapeutic effect of IL‐3, a single in situ injection of recombinant IL‐3 was administered immediately following SCI in mice (Figure 6A). The results showed that BODIPY^+^ lipid droplets were significantly reduced in the spinal lesion core at 28 dpi after IL‐3 injection compared to the control group (Figure 6B–D). Furthermore, there was a notable decrease in the area occupied by MAC2^+^ and CD68^+^ inflammatory cells in the IL‐3‐treated group at 28 dpi compared to the control group (Figure 6B,C,E,F). These results indicate a direct role of IL‐3 in promoting the clearance of lipid droplets by macrophages and mitigating inflammation in the lesion core after SCI.

### 3.6 In Situ Injection of IL‐3 Promotes Axon Regeneration, Neuronal Preservation and Locomotor Function Recovery After SCI

**FIGURE 7:**
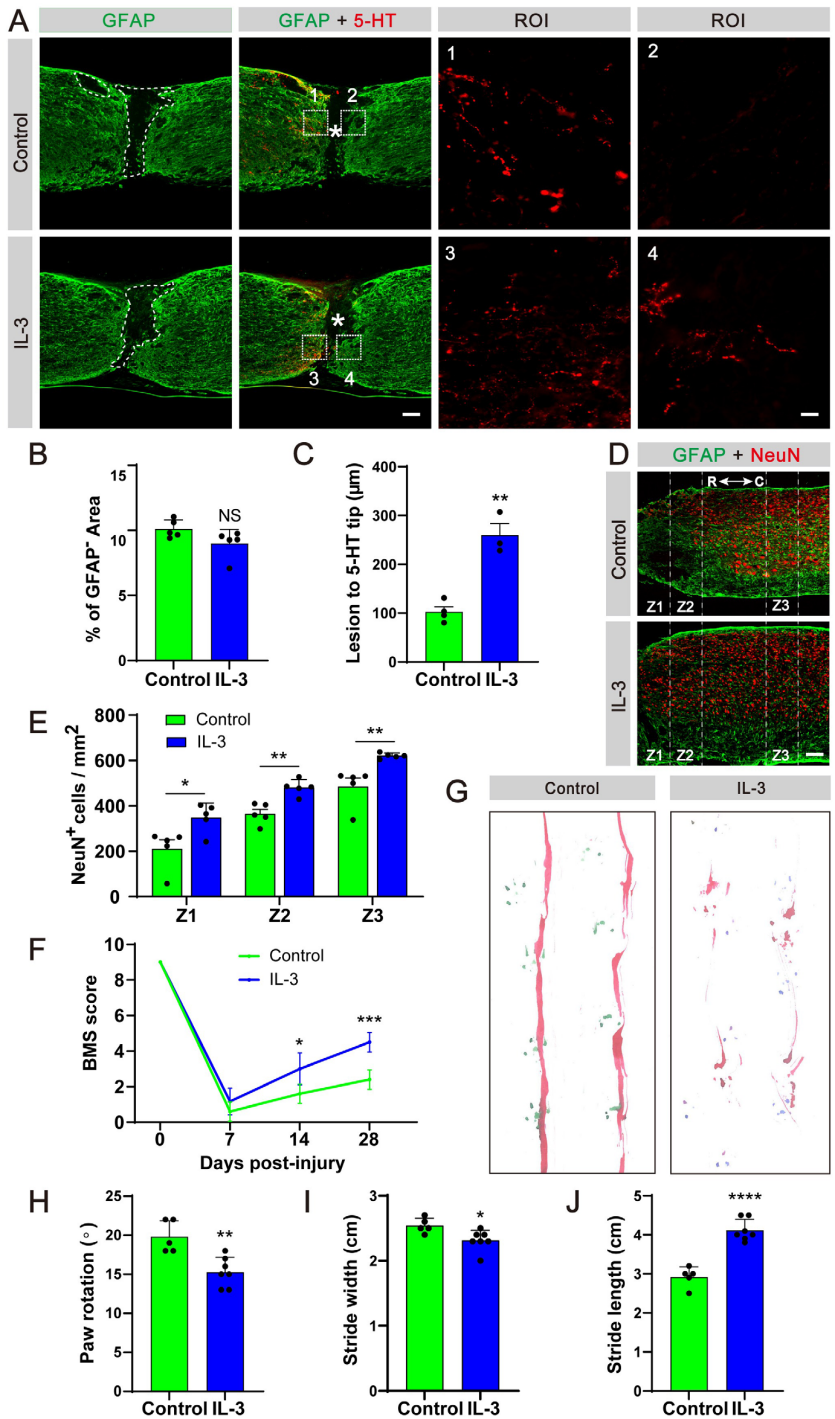
In situ injection of IL‐3 promotes neuronal survival and motor function recovery after SCI. (A) Immunofluorescent staining of GFAP (green) and 5‐HT (red) in sagittal sections of the control and IL‐3 groups at 28 dpi. The region of interest (ROI) represents the boxed region on the left and shows the astrocytic scar boundary. Asterisks indicate the injured core. (B) Quantification of the percentage of GFAP^−^ area in the spinal cord segment spanning the injured core at 28 dpi. (C) Quantification of the lesion distance to the 5‐HT axon tip. (D) Immunofluorescent staining of GFAP (green) and NeuN (red) in sagittal sections of the control and IL‐3 groups at 28 dpi. Scale bars: 200 μm (left panel in A) and D, 20 μm (right panel in A). (E) Quantification of NeuN^+^ cells in Z1–Z3 zones adjacent to the lesion core at 28 dpi. IL‐3 compared with control, *p < 0.05 and **p < 0.01 by Student's *t* test, *n* = 5 animals per group. (F) Locomotor function was evaluated by BMS before SCI and at 7, 14, and 28 dpi. *p < 0.05 and ****p < 0.0001 versus control group by two‐way ANOVA followed by Bonferroni's post hoc test, *n* = 7 animals per group. (G) Representative images of footprint analysis in the control and IL‐3 groups at 28 dpi. The front paws are shown in green dyes, and the hind paws are shown in red dyes. (H–J) Quantification of the paw rotation, stride width and stride length at 28 dpi. *p < 0.05, **p < 0.01 and ****p < 0.0001 by Student's *t* test, *n* = 5 animals per group.

We also conducted an investigation into the effects of IL‐3 treatment on lesion contraction, axon regeneration, neuronal preservation and locomotor function following SCI. While no significant differences in the extent of injury were observed between mice treated with IL‐3 and the control group (Figure 7A,B), immunofluorescent staining revealed a higher presence of 5‐HT axonal fibers at the injury site in mice receiving IL‐3 at 28 dpi compared to the control group (Figure 7A,C). Additionally, there was a notable increase in the number of surviving NeuN^+^ neurons in the Z1–Z3 region of the spinal cord in IL‐3‐treated mice compared to control mice (Figure 7D,E). Moreover, mice injected with IL‐3 displayed improved hind limb motor function at 7, 14 and 28 dpi, as evidenced by higher BMS scores (Figure 7F). Footprint analysis further confirmed that mice receiving IL‐3 exhibited greater locomotor function at 28 dpi (Figure 7G–J). In conclusion, our results demonstrate that IL‐3 treatment contributes to axon regeneration.

## 4 Discussion

**FIGURE 8:**
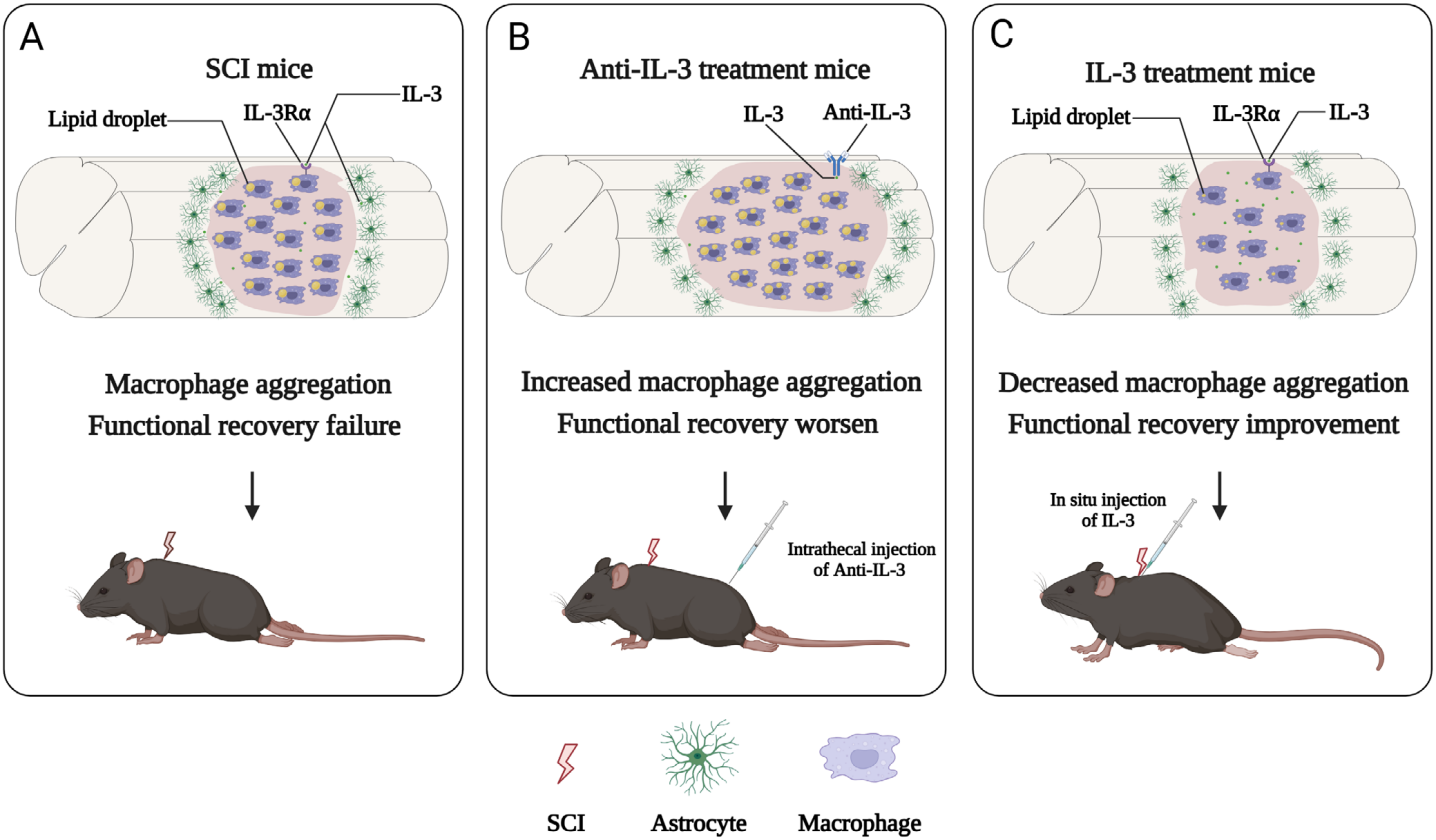
IL‐3 modulates macrophage phagocytic activity and promotes SCI repair. (A) After SCI, IL‐3 is secreted by astrocytes surrounding the lesion core, while foamy macrophages filled with lipid droplets gather in the injury center, contributing to the failure of functional recovery in mice. (B) Blockage of IL‐3 leads to increased aggregation of macrophage and lipid droplets at the injury core, resulting in the expansion of the lesion area and worsened locomotor function in mice. (C) Administration of IL‐3 results in a significant decrease in macrophage accumulation and promotes the clearance of lipid droplets by macrophages at the injury site, accompanied by enhanced functional recovery.

Despite the established pivotal role of various cytokines in SCI [31, 32, 33], the specific contribution of IL‐3 within this context remained unexplored prior to our investigation. Our study elucidates a crucial role of IL‐3/IL‐3Rα signaling pathway in modulating macrophages during the pathological progression of SCI. We observed a significant upregulation of IL‐3, predominately distributed around the injury core after SCI, with astrocytes identified as the primary source. Concurrently, IL‐3 was found to augment macrophage‐mediated lipid droplet clearance. In addition, this cytokine was revealed to inhibit the inflammatory response, reduce the lesion size, promote axonal regeneration, and support neuronal preservation (Figure 8). Together, these processes result in an improvement in locomotor function following SCI, emphasizing the therapeutic potential of regulating IL‐3Rα signaling as a strategy to ameliorate outcomes in SCI.

IL‐3 is a member of the β common chain (βc) cytokine family, alongside IL‐5 and granulocyte‐macrophage colony‐stimulating factor (GM‐CSF). These cytokines are characterized by their capacity to bind to specific α‐receptor subunits, while utilizing a common β‐receptor subunit to facilitate signal transduction, predominantly via the Janus kinase/signal transducer and activator of transcription pathway [13, 34, 35]. These βc cytokines, including IL‐3, GM‐CSF, and IL‐5, play a substantial role in regulating a broad spectrum of inflammatory responses, both accelerating pathogen clearance and potentially contributing to the pathogenesis of chronic inflammation [13]. IL‐3, in particular, demonstrates high‐affinity binding to the IL‐3Rα receptor, subsequently associating with the βc subunit. The signaling elicited by IL‐3 instigates a multitude of biological responses, including cell survival, proliferation, differentiation, migration, and effector functions across a wide array of immune and non‐immune cells [36]. Although correlations have been drawn linking IL‐3 to the pathophysiology of experimental autoimmune encephalomyelitis, multiple sclerosis, and Alzheimer's disease [15, 21, 22], the precise function of IL‐3 within CNS injury, particularly in the context of SCI, remains largely unexplored. In the present study, we document a substantial upregulation of IL‐3 expression following SCI, peaking at 14 dpi and persisting until 28 dpi. We identified astrocytes as the primary source of IL‐3 in the area surrounding the lesion epicenter. Furthermore, macrophages responsive to IL‐3 exhibited an upregulation in the expression of IL‐3Rα. This evidence collectively suggests that astrocyte‐derived IL‐3 plays a significant role in the pathophysiology of SCI.

Myelin debris generated subsequent to SCI contains neurite outgrowth inhibitors that obstruct axonal regeneration and remyelination. These myelin‐associated inhibitors exert a negative influence on the cytoskeleton in the growth cones of axotomized axons, including myelin‐associated glycoprotein, Nogo‐A, oligodendrocyte myelin glycoprotein, and ephrin‐B3 [37]. The alteration in shape and stability of growth cones triggered by these nerve growth inhibitory factors results in further degeneration. Moreover, myelin debris can also function as an inflammatory stimulus, inciting complement receptor 3‐dependent pro‐inflammatory responses in SCI [4]. Thus, macrophages, via phagocytosis of myelin debris, can foster an environment conducive to axonal regeneration. However, subsequent to the phagocytosis of myelin debris in SCI, lipids persist within macrophages due to imbalances in autophagy or lipid efflux mechanisms. This excessive intracellular accumulation of lipids leads to a disruption in intracellular lipid homeostasis and ultimately results in the formation of foamy macrophages [37]. These foamy cells have been frequently observed from 1 week post‐SCI [1]. A transcriptomics study in mice further revealed that following SCI, and more specifically, 7 dpi, macrophages augment their lipid catabolism, thereby exhibiting foamy cell characteristics [2]. The number of foamy macrophages has also been observed to increase over time, particularly at the lesion area, where they persist for a minimum of 4 weeks post‐SCI [1]. Therefore, despite the initial beneficial effects of myelin phagocytosis, the excessive uptake of myelin debris is a key factor driving the detrimental foamy macrophage phenotype [38, 39, 40].

In vitro and in vivo studies have demonstrated that toll‐like receptor 4, a critical receptor for the phagocytic of macrophages, induced by Lipopolysaccharide or the non‐toxic vaccine adjuvant E6020, promotes the phagocytosis of myelin debris, leading to an increase in spared and remyelinated axons. However, the impact on functional recovery remains unexplored [41, 42, 43]. Other reports have revealed that depletion of CD36, a scavenger receptor, in macrophages results in reduced lipid uptake and foamy cell formation, leading to smaller lesions and improved functional recovery after SCI [2, 44]. Our previous studies focused on the ATP‐binding cassette sub‐family A member 1 and its role in promoting lipid droplet efflux. We found that the use of D‐4F, an apolipoprotein A‐I peptidomimetic, can facilitate the removal of myelin debris and decrease the formation of foamy macrophages at the lesion core after SCI [45]. In the current study, we observed a significant increase in lipid droplet accumulation within macrophages treated with anti‐IL‐3 compared to those receiving the isotype IgG control. This suggests that IL‐3 plays a facilitating role in lipid droplet clearance post‐SCI. To validate this hypothesis, we administered IL‐3 locally after inducing SCI. The results demonstrated a notable reduction in lipid droplet accumulation within macrophages at the injury epicenter in IL‐3‐treated mice compared to the control group. These findings further support the notion that IL‐3 enhances the clearance of lipid droplets by macrophages in the context of SCI.

Recent studies have uncovered that osteopontin, a matricellular glycoprotein produced by hematogenous macrophages, induces extension of astrocyte processes towards the infarct border zone, potentially aiding in repair of the ischemic neurovascular unit [46]. Additionally, transforming growth factor‐beta secreted by M2 macrophages may trigger the conversion of astrocytes to the A2 phenotype, which has neuroprotective properties and promotes tissue repair and regeneration following SCI [47]. These findings suggest that the reciprocal communication between macrophages and astrocytes significantly influences neuroinflammation. However, the precise mechanisms by which astrocytes regulate macrophage activity after SCI remain unclear. In our current investigation, we have uncovered a previously unrecognized pathway in which IL‐3, secreted by astrocytes, modulates macrophage activity to enhance the clearance of lipid droplets after SCI. When IL‐3 was inhibited using an IL‐3‐neutralizing antibody, we observed a notable increase in the number of foamy cells, accompanied by an expansion in the area covered by inflammatory cells and a corresponding enlargement in lesion size. Conversely, mice that received an in situ injection of IL‐3 exhibited a reduced presence of foamy cells and a decrease in the area occupied by inflammatory cells. However, we did not observe a significant reduction in lesion size in the IL‐3 injected mice. We propose that astrocytes secrete sufficient IL‐3 to promote injury healing after SCI, and excessive secretion of IL‐3 from astrocytes may mask the efficacy of a single dose of injected IL‐3.

In this study, we utilized specific markers such as MAC2 and F4/80 to identify macrophages within the subjects of our research. However, this approach is insufficient for distinguishing infiltrating macrophages from activated resident microglia in the context of SCI. We acknowledge the potential for confusion and the limitations this may impose on the generalizability of our findings. Furthermore, our current study does not provide direct evidence linking the observed phenotypic changes to IL‐3 secreted by astrocytes. To address this issue, we propose using transgenic mice with an astrocyte‐specific knockout of IL‐3 in future studies. Additionally, the definitive molecular mechanisms by which IL‐3 mediates lipid clearance in macrophages remains to be elucidated.

In summary, our findings demonstrate a significant upregulation of IL‐3 distributing around the injury core after SCI, with astrocytes identified as the primary source. Activation of the IL‐3/IL‐3Rα pathway promotes lipid droplet clearance, resolution of inflammation, lesion shrinkage, neuronal preservation, axon regeneration, and ultimately, leads to motor function recovery after SCI. Our study highlights the beneficial role of IL‐3 in regulating macrophage phagocytic activity and promoting SCI repair, providing new evidence to decipher the cellular interactions in SCI pathology.

## Author Contributions

J.J., L.C. and M.Z. designed the study and carried out the project administration. J.L., F.Y., J.Y. and J.H. performed the experiments. Y.Z., J.W., F.S., Z.L., S.Y., F.Y. and D.T. analyzed the data. J.L., M.Z. and L.C. contributed to writing and revising the manuscript. The authors read and approved the final manuscript.

## Ethics Statement

All experiments involving animals were approved by the Ethics Committee of Anhui Medical University (Approval No. LLSC20231692).

## Conflicts of Interest

The authors declare no conflicts of interest.

## Supporting information


Data S1.

## Data Availability

All data generated or analyzed during this study are included in this published article.
