# Supplementary material for: Interleukin‐3 Modulates Macrophage Phagocytic Activity and Promotes Spinal Cord Injury Repair
Source: CNS Neurosci Ther. 2024 Dec 19;30(12):e70181. doi: 10.1111/cns.70181 (PMC11656101; doi:10.1111/cns.70181)
Supplement: Supplementary file 1 — Data S1. [file CNS-30-e70181-s001.docx]

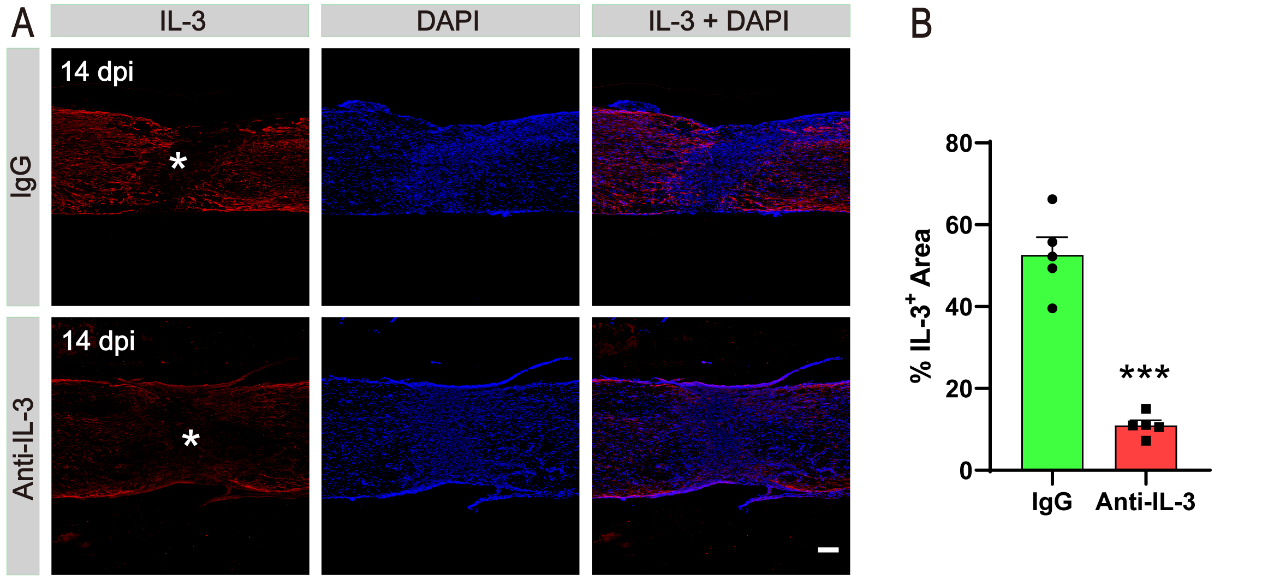


**Supplementary Figure.1** Intrathecal injection of IL-3 neutralization antibody decreases IL-3 level in the injured spinal cord at 14 dpi. **A** Immunofluorescent staining of IL-3 (red) and DAPI (blue) in sagittal sections of the IgG and Anti-IL-3 groups at 14 dpi. Asterisks indicate the injured core. Scale bar: 200 μm. n = 5 animals per group. **B** Quantification of the percentage of IL-3^+^ area in the spinal cord segment spanning the injured core at 14 dpi. ^***^P < 0.001 by Student’s t test.

**
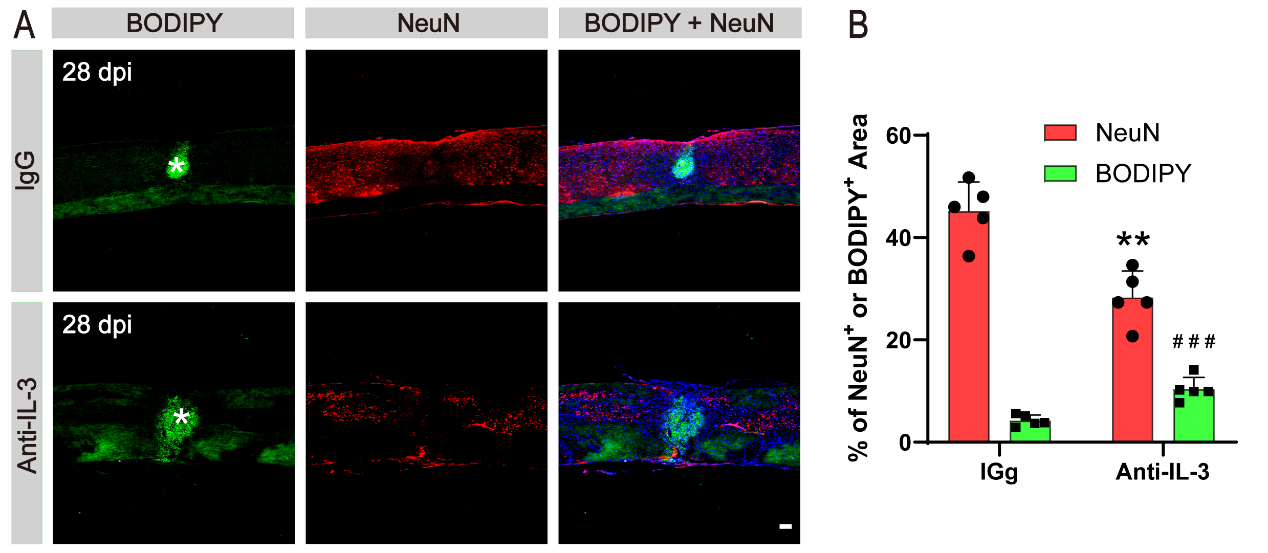
**

**Supplementary Figure.2** Intrathecal injection of IL-3 neutralization antibody results in an increase in BODIPY^+^ signals and a decrease in NeuN^+^ signals in the injured spinal cord at 28 dpi. **A** Immunofluorescent staining of BODIPY (green), NeuN (red) and DAPI (blue) in sagittal sections of the IgG and Anti-IL-3 groups at 28 dpi. Asterisks indicate the injured core. Scale bar: 200 μm. n = 5 animals per group. **B** Quantification of the percentage of NeuN^+^ or BODIPY^+^ area in the spinal cord segment spanning the injured core at 28 dpi. IGg group compared to Anti-IL-3 group ^**^P < 0.01 by Student’s t test and ^###^P < 0.001 by Student’s t test.


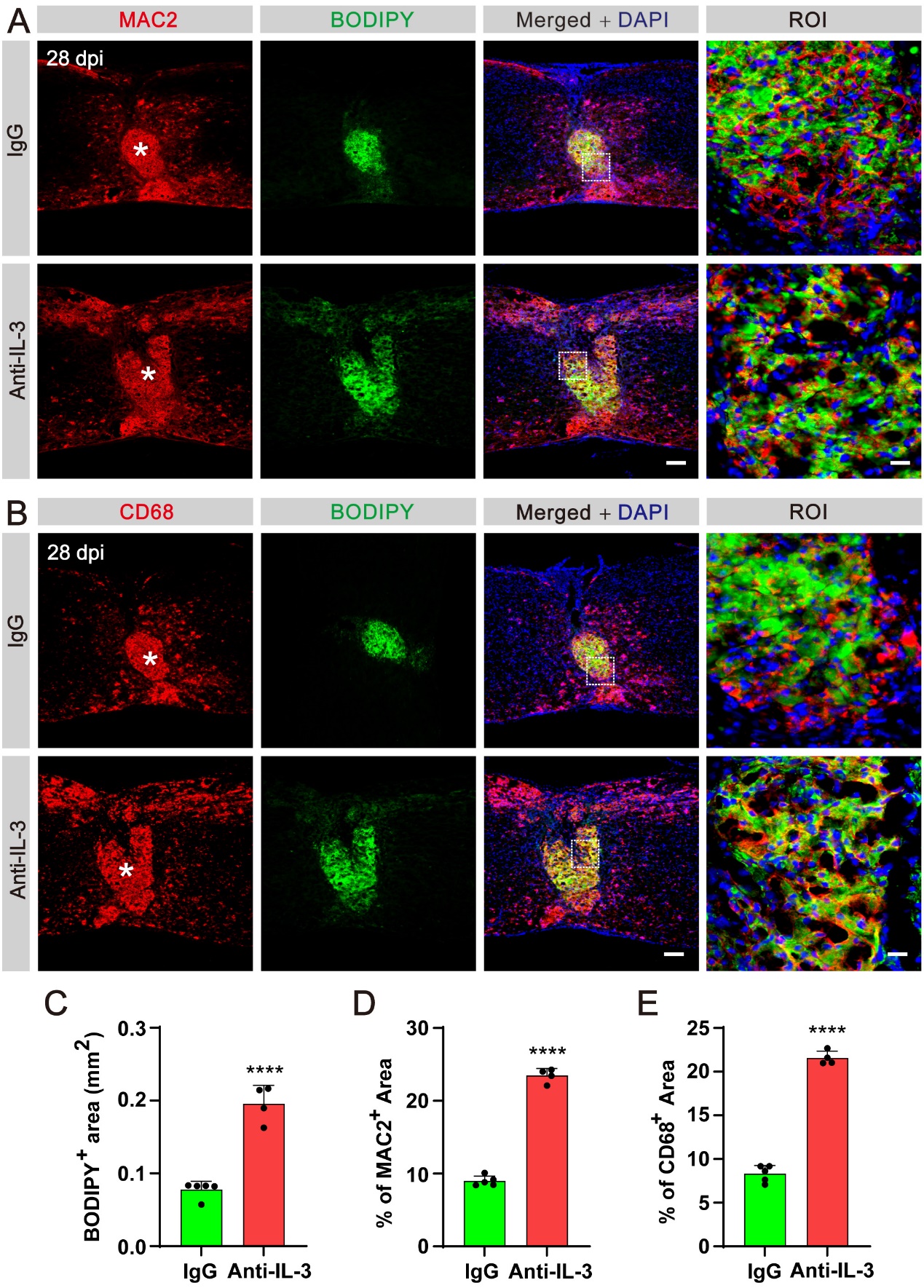


**Supplementary Figure.3** Intrathecal injection of IL-3 neutralization antibody hinders clearance of lipid droplets after SCI. **A** Immunofluorescent staining of MAC2 (red) and BODIPY (green) in sagittal sections of the IgG and Anti-IL-3 groups at 28 dpi. **B** Immunofluorescent staining of CD68 (red) and BODIPY (green) in sagittal sections of the IgG and Anti-IL-3 groups at 28 dpi. The region of interest (ROI) represents the boxed region on the left. Asterisks indicate the injured core. Scale bars: 200 μm (left panel in A, B) and 20 μm (right panel in A, B). n = 4-5 animals per group. **C** Quantification of the BODIPY^+^ area in the spinal cord segment spanning the injured core at 28 dpi. **D** and **E** Quantification of the percentage of MAC2^+^ area (**D**) and CD68^+^ area (**E**) in the spinal cord segment spanning the injured core at 28 dpi. ^****^P < 0.0001 by Student’s t test.
